# Research-Based Intervention (RBI) for Autism Spectrum Disorder: Looking beyond Traditional Models and Outcome Measures for Clinical Trials

**DOI:** 10.3390/children9030430

**Published:** 2022-03-18

**Authors:** Antonio Narzisi, Yurena Alonso-Esteban, Gabriele Masi, Francisco Alcantud-Marín

**Affiliations:** 1 Department of Child Psychiatry and Psychopharmacology, IRCCS Stella Maris Foundation, 56812 Pisa, Italy; gabriele.masi@fsm.unipi.it; 2 Department of Developmental and Educational Psychology, University of Valencia, Avenida de Blasco Ibáñez, 13, 46010 Valencia, Spain; yurena.alonso@uv.es (Y.A.-E.); francisco.alcantud@uv.es (F.A.-M.)

**Keywords:** Autism Spectrum Disorder, intervention, outcome, children, precision medicine

## Abstract

The rising prevalence of Autism Spectrum Disorders (ASD) has led to a quickly increasing need for effective interventions. Several criteria and measures have been developed to critically assess these interventions with particular focus on the evaluation of the efficacy. Given the huge diversity of ASD symptoms and the different levels of severity across individuals, identifying a one size fits all intervention approach is challenging, and the question What works and for whom? Remains still unanswered. Why do we seem to be dragging our feet on this fundamental issue? The main aim of this paper is to answer this question through four non-alternative points. First, there are a scarce number of studies with a solid methodology. Secondly, most trials on intervention efficacy for ASD are designed exclusively in terms of behavioral outcomes. Thirdly, there is a reduced use of biologically oriented outcome measures. Fourthly, in most clinical trials, appropriate practices emerging from research evidence are not systematically applied. A strong effort to improve the methodology of clinical trials is mandatory for the future of autism research. The development of a research-based intervention (RBI) perspective aimed at better integrating: (a) evidence-based approaches; (b) more sensitive behavioral outcome measures; and (c) biomarkers, with the aim of increasing a more detailed clustering of phenotypes, may strongly improve our approach to a precision medicine.

The rising prevalence of Autism Spectrum Disorders (ASD) [1] has led to a quickly increasing need for effective interventions. Timely interventions aim to prevent or minimize the developmental effects of early impairments [2]. Currently, a large number of methods and techniques are available, with different levels of scientific evidence [3,4]. Several criteria and measures have been developed to critically assess these interventions [5,6,7,8,9,10], with particular focus on the evaluation of the efficacy [11,12,13,14,15]. However, despite the rapidly growing number of studies, most of them fall below the threshold of adequate methodological quality in conformity with the Cochrane risk of bias tool [16]. It is organized into a definite set of domains of bias, focusing on different facets of trial design, conduct, as well as reporting the risk of bias in RCT contained in Cochrane Reviews.

Given the huge diversity of ASD symptoms and the different levels of severity across individuals, identifying a “one size fits all” intervention approach is challenging, and the question “What works and for whom?” [15] remains still unanswered. In other words, in this field we are still far from the target of “precision medicine”. Why do we seem to be dragging our feet on this fundamental issue? The main aim of this paper is to answer this question through four non-alternative points:

First, as previously reported, there are a scarce number of studies with a solid methodology, and this issue strongly limits the generalization of the findings [16]. These limits, in line with the Cochrane risk of bias tool, can be summarized as follows: (a) small sample size; (b) small number of multi-center studies; (c) the absence of long-term research on outcome effects; (d) the reduced adaptation of some evidence-based interventions outside of the country and research group in which they were developed; and (e) the excessive number of novel intervention models. It is emblematic that, currently, only 12% of published papers meet the criteria for high methodological quality [16]. This means that research in the arena of intervention in ASD, apart from a few rare examples [9,17], remains below cutting-edge levels [18,19].

Secondly, (a) most trials on intervention efficacy for ASD are designed exclusively in terms of behavioral outcomes. Despite the wide range of psychometric measures providing useful information (e.g., about IQ, language), they cannot assess subtle changes in the quality of the core symptomatology of the disorder (e.g., parent–child interaction, social-communication behaviors); (b) the differences in the behavioral outcome measures used by studies do not allow for comparison between trials.

Over the years, the low reliability of outcome measures and the lack of objective markers to identify subgroups of young children with different levels of response to specific interventions has prompted researchers to develop more sensitive methodological designs. The systematic adoption of instruments aimed at studying subtle changes in children and in parent–child interactions may be helpful at further improving current outcome measures. Among the well-conducted research in ASD [20,21] the ADOS-BOSCC (Brief Observation of Social Communication Change) [19] was drawn up specifically as an outcome measure in early intervention trials, to detect subtle clinical changes especially in the Social Communication domain (e.g., eye contact, facial expressions, gestures, vocalizations, social overtures, social responses, requesting and engagement) [18,22]. The ADOS-BOSCC is based on videotaped observation of parent–child naturalistic social interactions and free play [19]. Findings about ADOS-BOSCC: (a) support it as a promising test for recordings modifications in social communication behaviors as a result of a behavioral treatment [23]; and (b) indicate strong reliability and validity also in children with ASD who have reduced use of language. Findings in addition claim that the ADOS-BOSCC may be more sensitive in detecting subtle changes in social communication, compared to other instruments [22]. Unfortunately, due to time-consuming, training and costs, few trials have used ADOS-BOSCC as a supplementary outcome measure [21,24].

The findings of a seminal research carried out by Green et al. in 2010, replicated in 2016 [25], showed that the DCMA (Dyadic Communication Measure for Autism) could be used to study, from a punctiform perspective, the parental child interaction. [12,26]. It provides a reliable and naturalistic assessment of the interactive exchanges between parent and child in a play setting [12,26,27]. The interaction is then coded from video-tapes, and three levels are observed: (a) parent synchrony and responsiveness; (b) child communicative initiations, responses, and communicative functions; (c) amount of mutual shared attention between parent and child [12,26,27]. However, these measures of parent–child interactions are not yet systematically included in most clinical trials, with few exceptions [28,29].

Thirdly, a reliable response to the question about what works and for whom is difficult, given the reduced use of biologically oriented outcome measures. In recent years, biomarker research has become an increasing goal in the field of ASD. Although much effort is being made, there are still no reliable and valid biological or brain imaging markers that would allow us to study subtle changes more objectively during the intervention [30,31,32].

To address the question, it would be essential to implement studies including, alongside behavioral outcome measures, also biologically oriented outcome measures, such as EEG, eye-tracking, f-MRI, and wearable devices for neurovegetative parameters. Integrating the results of biological and behavioral outcome measures using artificial intelligence may help to identifying subtypes of ASD with different responses to specific interventions, with the aim to develop and monitor specific biological therapies [31].

In terms of biological-oriented outcome measures, the research has shown that visual patterning, explored through eye-tracking, could be a promising measure to monitor subtle response to the intervention, to predict outcomes, and to determine unique features of the child’s performance that fit with the proposed mechanisms of change [33]. Over the past two decades, eye-tracking device has been widely applied in ASD research [34], namely, to study joint attention [35], social attention [36,37], visual preference [38], responses to dyadic bids [39], theory of mind abilities [40], facial expression recognition [41], and attentional preferences at both the semantic [42] and perceptual levels [43].

Since the literature has identified unordinary models of functional brain connectivity in young children with ASD based on electrophysiological measures [44,45,46], the EEG and fMRI should also be more closely considered in trials using biologically oriented outcome measures. Studies from animal models have reported multiple atypicality in functional brain connectivity, known as connectopathy [46,47]. Zerbi et al. carried out a cross-etiological investigation of fMRI-based connectivity in the mice, showing that different ASD-associated etiologies cause a broad spectrum of connectional abnormalities, with different, often diverging, connectivity signatures [47]. These findings suggest that etiological variability is a key determinant of connectivity heterogeneity in ASD, accounting for conflicting findings in different clinical populations.

Following the wave of atypical connectivity, postmortem studies have revealed increased density of excitatory synapses in the brain of individuals with ASD, with a putative link to aberrant mTORdependent synaptic pruning [46,47]. These observations raise the question of whether an excess of synapses may cause aberrant functional connectivity in ASD. Using resting state fMRI, electrophysiology and in silico modelling in Tsc2 haploinsufficient mice, Pagani et al. showed that mTOR-dependent increased spine density is associated with ASD-like stereotypies and cortico-striatal hyperconnectivity [47]. These deficits are completely rescued by pharmacological inhibition of mTOR. Pagani et al. showed that the identified transcriptomic signature is predominantly expressed in a subset of children with ASD, thereby defining a segregable autism subtype [47]. The findings of Pagani et al. causally link mTOR-related synaptic pathology to large-scale network aberrations, revealing a unifying multi-scale framework that mechanistically integrates developmental synaptopathy and functional hyperconnectivity in ASD. Thus, the identification of etiologically relevant connectivity subtypes could improve diagnostic label accuracy in the non-syndromic ASD population and paves the way for personalized treatment approaches.

In 2012, a trial by Dawson et al. [11] showed that the behavioral changes in preschoolers with ASD after a Naturalistic Developmental Behavioral Intervention (NDBI) [17] were associated with the normalization of brain activity patterns, parallel to the improvements in social behavior.

In 2016, Yang et al. demonstrated that functional MRI may predict responses to evidence-based behavioral intervention [48]. In their study, Yang et al. [48] identified neural predictors of pre-intervention activity levels in response to biological versus scrambled movement in neural circuits that support social information processing and social motivation/reward. The predictive value of their findings in ASD children was supported by cross-validated multivariate pattern analysis. The implications of these findings are far-reaching and should greatly accelerate progress towards more accurate and effective interventions for the core deficits of ASD [48].

Fourthly, in most clinical trials, appropriate practices emerging from research evidence are not systematically applied. Following the advices of Zwaigenbaum et al. [49], effective intervention research and practice should: (a) include naturalistic behavioral and developmental interventions; (b) involve parents in the treatment setting; (c) act on secondary and core ASD deficits; (d) take into account the socio-cultural facets of families involved in treatment and their affordability as possible variables moderating outcomes; (e) include subjects come from many different countries to evaluate how factors of cultural affiliation might affect therapeutic compliance; (f) apply a rigorous research methodology and be faithful to the implementation of the model; (g) examine active ingredients of effective treatments such as treatment hours; and (h) use a standardized outcome assessment protocol.

In addition to the above suggestions, another best practice aimed to capture subtle changes in children and their interacting families is the technique of video-feedback [12,50]. It typically consists of videotaped play sessions between parent and child. These recordings are then observed by the parent along with a clinician. The role of the clinician is to help the parent reflect on the child’s behaviors as well as the parent’s own behaviors. The goal of video-feedback is to support the parent in being able to pick up on the child’s subtle communicative cues and develop effective interaction strategies [46]. Literature showed that the video-feedback can be integrated into parent mediated interventions to increase the child’s language skills [51], make parents less stressed and more competent in their role [52], increase parental synchronous acts [12], reduce, in the long term, autistic symptomatology [24], and decrease parental asynchronous acts [52].

Although substantial advancement continues to be made within the past decade in the study of ASD, the translational research about the efficacy and effectiveness of interventions is hampered by extreme heterogeneity in models, as well as in outcome measures. These limits do not allow for an adequate generalization of findings and a significant progress in precision medicine. The lack of a systematic integration of behavioral and biomarker outcomes in clinical trials targeting the core symptomatology of ASD decrease our potential for new knowledge (i.e., from pharmacological studies) [53]. A strong effort to improve the methodology of clinical trials is mandatory for the future of autism research. As reported by Ruggeri et al. [54], recognized biomarkers, neuropsychological assessments, electrophysiological measurements, and functional brain imaging will be linked with novel biomarkers identified from omics data (e.g., proteomic, epigenomic) to generate multimarker panels. Management of these data and analysis methods using artificial intelligence techniques will be central to identifying these biomarker panels.

## Conclusions

The development of a research-based intervention (RBI) perspective aimed at better integrating: (a) evidence-based approaches; (b) more sensitive behavioral outcome measures; and (c) biomarkers, with the aim of increasing a more detailed clustering of phenotypes, may strongly improve our approach to a precision medicine. This target could help us to better identify the most effective intervention solution for children and improve and accelerate the development of effective treatments (including innovative drugs) for the core deficits of ASD.

## Data Availability

There are no data in this paper.
